# Addition of Coriander during Fermentation of Korean Soy Sauce (Gangjang) Causes Significant Shift in Microbial Composition and Reduction in Biogenic Amine Levels

**DOI:** 10.3390/foods9101346

**Published:** 2020-09-23

**Authors:** Mohamed Mannaa, Young-Su Seo, Inmyoung Park

**Affiliations:** 1 Department of Integrated Biological Science, Pusan National University, Busan 46241, Korea; mannaa_mohamed@yahoo.com; 2 Department of Plant Pathology, Cairo University, Giza 12613, Egypt; 3 Department of Oriental Food and Culinary Arts, Youngsan University, Busan 48015, Korea

**Keywords:** soy sauce, microbiome, coriander, *Chromohalobacter*

## Abstract

The microflora of Korean soy sauce (gangjang) play an important role in maintaining its quality and safety. Hence, it is important to study the microflora and the possible approaches to improve their composition. In this study, the effect of adding coriander during soy sauce fermentation on the microflora and biogenic amines was evaluated using metagenomics and ^1^H NMR analyses, respectively. The β-diversity showed a clear distinction between the microbiota of the coriander and control groups. Microbial composition analysis revealed noticeable shifts, as Firmicutes abundance was significantly higher in the coriander group (91.77%) than that in the control (38.78%). The dominant bacterial family in the coriander group was the Bacillaceae (57.94%), while Halomonadaceae was dominant in the control group (49.77%). At the species level, *Chromohalobacter beijerinckii* dominated the microbial community in the control group (49.54%), but not (4.43%) in the coriander group. Moreover, there was a negative correlation between the Bacillaceae and several other bacterial families, including Halomonadaceae, which indicated a possible antagonism and partly explained the reduction in *Chromohalobacter* abundance in the coriander group. The levels of the biogenic amines histamine, putrescine, and tyramine, which are considered potential health risk factors, were significantly lower in the coriander soy sauce than those in the control sauce. The results of this study suggest that the addition of coriander during Korean soy sauce fermentation is beneficial, as coriander significantly reduces the levels of biogenic amines and the bacteria that produce them.

## 1. Introduction

Soy sauce is the most popular fermented soybean product, owing to its distinct intense umami taste; it is mainly used as a flavoring condiment worldwide [1]. In East Asia, each country has its unique soy sauce, which slightly differs based on the traditional method of preparation and the ingredients used [2]. In Korea, traditional fermented soy sauce (gangjang) is prepared from soybean blocks (meju), which are fermented by leveraging the fungal and bacterial populations naturally occurring in the raw materials. The dry, moldy blocks are then soaked in brine solution (~20% salt) for a second long-term fermentation that produces a solid component, doenjang, and a liquid component, gangjang [3]. An array of aromatic and flavoring compounds, as well as several bioactive compounds, have been detected in soy sauce [4]. Previous reports have indicated the potential health benefits of soy sauce, including a preventive effect on thrombus formation, as well as antioxidant, antitumor, antimicrobial activities [5,6,7,8].

The quality and properties of fermented soy sauce depend—for the most part—on the microbial composition during fermentation. As this fermentation is conducted under non-sterile conditions and relies on the spontaneous growth of bacteria and fungi, it is not surprising that there are huge variations in the composition of fermented soy sauce microbiota. Variations in the microbial composition of soy sauce may result in the following: (1) differences in the organoleptic properties and (2) the possible generation of undesirable metabolites, such as biogenic amines or toxins [9,10]. Biogenic amines are low-molecular weight organic compounds that are found in fermented foods; they are generated as a result of amino acid decarboxylation or the amination and transamination of aldehydes and ketones by specific microbes [11,12]. The consumption of large amounts of biogenic amines can result in physiological and toxicological effects that may lead to poisoning; hence, the content of biogenic amines in fermented foods should be minimized [12]. Therefore, it is of great importance to study the microbial composition and factors that affect and shape the microbial profile of traditional soy sauce.

There are a few reports on the microbial composition of fermented soy sauce. In general, the brine fermentation step restricts the growth of several undesirable microbes and creates more favorable conditions for the growth of halophilic lactic acid microbes, which dominate in soy sauce and are mainly responsible for the flavor [10]. 16S rRNA sequence analysis has been used to study the microbial composition of Korean soy sauce; it has revealed *Halanaerobium, Tetragenococcus, Staphylococcus*, and *Bacillus* spp. to be the dominant microbes [9]. Recently, culture-independent techniques utilizing advanced high-throughput sequencing have been used for microbial community profiling of several fermented foods [13,14]. However, only a limited number of studies have used such an advanced approach to study the microbial composition of traditional Korean soy sauce [15,16]. A recent study showed that halotolerant and halophilic microbes are mainly responsible for soy sauce fermentation, and that they are derived from the sea salt added before the brine fermentation step, whereas the non-halophilic microbes derived from meju are abundant during the early fermentation stages [15].

Herbs and spices are well-known food additives (with potential health benefits) that preserve food and exhibit antioxidant activity by virtue of their high phenolic contents [17,18]. Coriander (*Coriandrum sativum* L.) is a member of the Umbelliferae family; this annual, herbaceous plant has culinary and medicinal uses [19]. Coriander leaves and seeds are rich sources of antioxidants, and contain volatile compounds that have been reported to inhibit the growth of a range of microorganisms [20,21]. Therefore, we hypothesized that the incorporation of coriander during soy sauce production via fermentation would have a significant impact on the microbial composition, possibly leading to a reduction in the levels of harmful metabolites. The objective of this study was to evaluate the effect of adding coriander during the fermentation of Korean soy sauce on the microbial composition and biogenic amine contents using high-throughput metagenomic sequencing and proton nuclear magnetic resonance spectroscopy (^1^H NMR), respectively.

## 2. Materials and Methods

### 2.1. Preparation of Soy Sauce Samples

Soy sauce samples were prepared using the traditional two-stage Korean method [3]. Briefly, dry cooked soybean blocks (meju) were firstly fermented using naturally occurring bacteria and fungi. The prepared fermented moldy meju blocks (~2 kg, Gigang County, Korea) were then soaked in 6 L of 20% (*w*/*v*) solar salt solution (Shinan, Korea) in porcelain containers. Pure charcoal (3 pieces; 3 cm × 3 cm × 10 cm) and dried red pepper (5 pieces) purchased from the local market (Gigang County) were added to the mixture. In the treatment samples, 200 g of fresh coriander was added to the mixture after trimming the roots. The mixture was then fermented for 45 days under sunlight; the lid of the pot was open by day and closed by night. The solid component, fermented soy paste (doenjang), was separated from the liquid component, soy sauce (gangjang); this was followed by boiling for 10 min. The soy sauce samples were then stored at 4 °C until they were used for metagenomic sequencing and analysis.

### 2.2. DNA Extraction, Sequencing, and Metagenomic Analysis

The collected soy sauce samples were first centrifuged for 15 min at 10,000× *g* and the obtained pellet was washed with sterile distilled water to remove excess water and salts. A part of the obtained pellet (250 mg) was used for metagenomic DNA extraction using a PowerSoil^®^ DNA Isolation Kit (MO BIO Laboratories, Carlsbad, CA, USA), based on the manufacturer’s protocol. The obtained DNA was checked for quality and concentration using agarose gel electrophoresis and a NanoDrop2000 spectrophotometer (Thermo Fisher Scientific, Wilmington, NC, USA). Qualified samples were stored in Tris-EDTA buffer at −20 °C until further analysis.

The hypervariable regions, V3 and V4, of the 16S rRNA gene were used for the metagenomic analysis of the obtained DNA samples. The conditions of PCR amplification and sequencing, which were performed in an Illumina^®^ MiSeq^®^ platform at Macrogen (Seoul, South Korea), were based on the protocol provided along with the Herculase II fusion DNA polymerase Nextera XT Index Kit V2. The following primer pair was used:

(F), 5′-TCGTCGGCAGCGTCAGATGTGTATAAGAGACAGCCTACGGGNGGCWGCAG-3′;

(R), 5′-GTCTCGTGGGCTCGGAGATGTGTATAAGAGACAGGACTACHVGGGTATCTAATCC-3′.

The paired-end reads obtained from sequencing were merged using the fast length adjustment of short reads (FLASH; http://ccb.jhu.edu/software/FLASH/) [22]. The Illumina adaptors and the short and low-quality reads were trimmed, and the raw sequences were purified using the Scythe (v0.994) (https://github.com/vsbuffalo/scythe) and Sickle programs (https://github.com/najoshi/sickle). Following purification, clustering and annotation were performed using the CD-HIT-OTU-MiSeq and UCLUST algorithms, and qualified sequences were organized into respective operational taxonomic units (OTUs) at a cut off value of 97%, using the Greengenes database [23,24,25]. Analysis of the microbiota of soy sauce samples—including diversity statistics and taxonomic assignments of the obtained OTUs from the phylum to the species level—was performed using the Quantitative Insights into Microbial Ecology version 2 (QIIME2) pipeline [26]. The obtained sequences were deposited as a sequence read archive in the National Center for Biotechnology Information database (Bethesda, MD, USA) under the BioProject ID PRJNA640944.

### 2.3. Quantification of Biogenic Amines Using ^1^H NMR Spectroscopy

Biogenic amines (i.e., histamine, putrescine, and tyramine) in the prepared Korean soy sauce were analyzed using ^1^H NMR spectroscopy as per a previously described method [27]. Briefly, 1 mL—from each 10-fold diluted supernatant—of each Korean soy sauce sample was freeze-dried and dissolved in 600 μL of 99.9% deuterium oxide containing 2 mM trimethylsilylpropanoic acid (Sigma-Aldrich, St. Louis, MI, USA) for standardization. The solutions were then transferred into NMR tubes, and their ^1^H NMR spectra were recorded using a Bruker Avance 500 MHz spectrometer (Bruker Biospin, Rheinstetten, Germany). Histamine, putrescine, and tyramine were identified and quantified using the profiler option in the Chenomx NMR suite program (v8.6, Chenomx Inc., Edmonton, AB, Canada).

### 2.4. Statistical Analysis

Statistical analysis of the bacterial microbiome was performed using QIIME2 scripts, R (version 3.1.3), and the PAleontological STatistics software package (PAST) version 3.23 [28]. Principle coordinates analysis (PCoA) was performed based on Bray–Curtis and Euclidean distances, for assessing the β-diversity between groups. Student’s *t*-test was used for statistical analysis of the relative abundance of bacterial taxa at different taxonomic levels, and *p*-values < 0.05 were considered significant. Pearson’s correlation analysis and correlogram plotting were performed using PAST.

## 3. Results

### 3.1. Microbial Diversity in Soy Sauce Samples Prepared with or without Coriander

High-throughput sequencing resulted in 1,374,263 reads with an average of 229,044 reads per sample; the total bases, read counts, GC%, Q20%, and Q30% for each sample are shown in Table S1. Following pre-processing and clustering using CD–HIT–OTU to remove low-quality reads and chimeras, 470,857 total reads were obtained with an average of 78,476 ± 19,598 reads per sample, ranging from a minimum of 48,207 to a maximum of 100,810.

**Figure 1:**
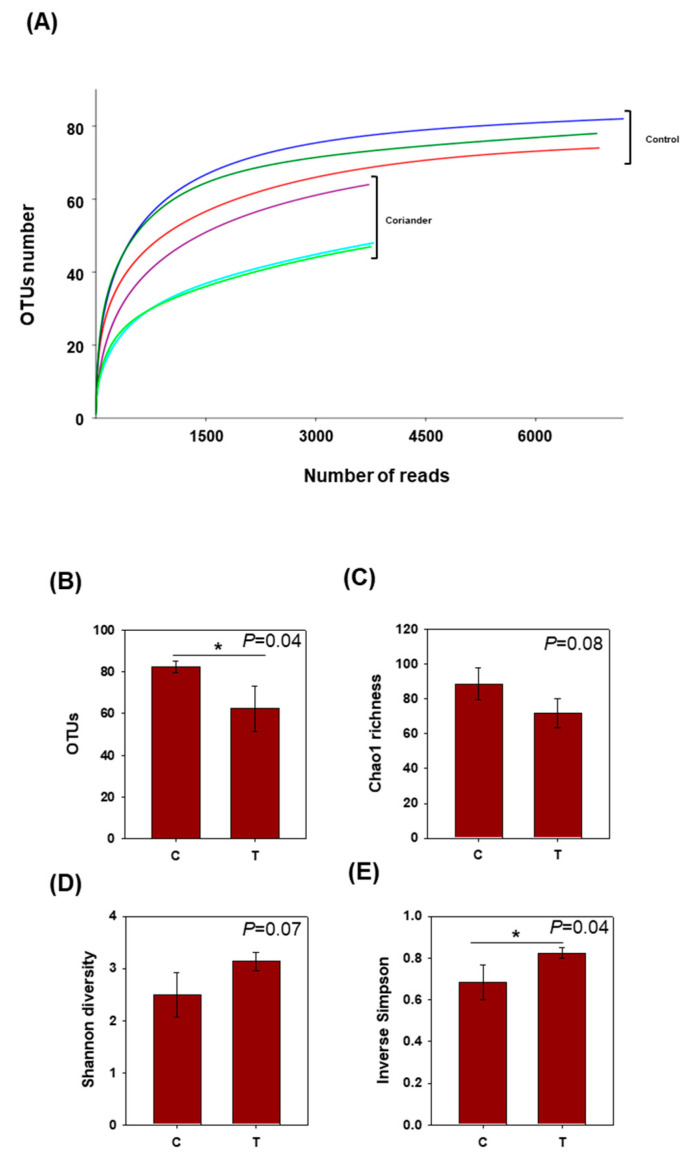
(**A**) Rarefaction curves of the obtained 16S rRNA sequence reads against the assigned operational taxonomic units (OTUs). Alpha diversity in soy sauce samples prepared by adding coriander, compared to that in control. (**B**) Number of OTUs, (**C**) Chao1 richness index, (**D**) Shannon diversity index, and (**E**) inverse Simpson’s index. Data represent means and standard deviations of three replicates for each soy sauce group. *: a significant difference at *p* < 0.05. C = control, T = coriander treatment.

Rarefaction analysis on the sequences obtained from both soy sauce groups indicated satisfactory sequencing depth, as a near plateau level was achieved at approximately 2000 reads, particularly for the control group (Figure 1A). The number of OTUs was significantly (*p* < 0.05) higher in the control soy sauce group compared to that in the coriander group (Figure 1B). There was no significant difference between both groups with respect to Chao1 richness and Shannon alpha diversity index (Figure 1C,D). However, the inverse Simpson index was significantly higher for the coriander soy sauce group compared to that for the control (Figure 1E).

**Figure 2:**
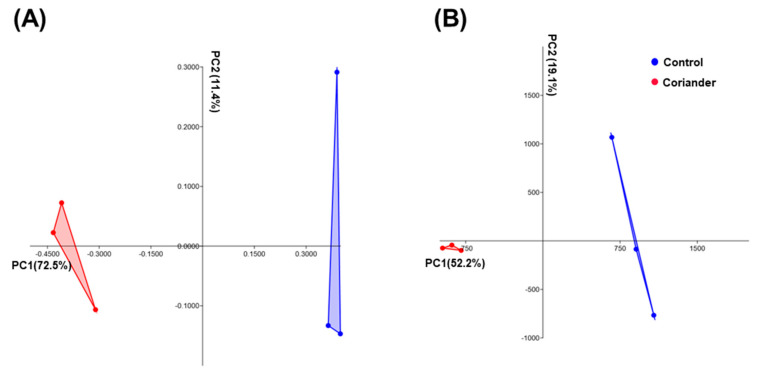
Principle coordinates analysis of (**A**) Bray–Curtis and (**B**) Euclidean distances of soy sauce samples prepared by adding coriander during fermentation, compared to those of control. Each dot represents a replicate from the different groups of soy sauce.

PCoA revealed an evident distinction between both soy sauce groups with respect to the β-diversity, as measured using the Bray–Curtis and Euclidean distance matrices (Figure 2A,B).

### 3.2. Microbial Structure and Dominant Taxa in Coriander and Control Soy Sauce

**Figure 3:**
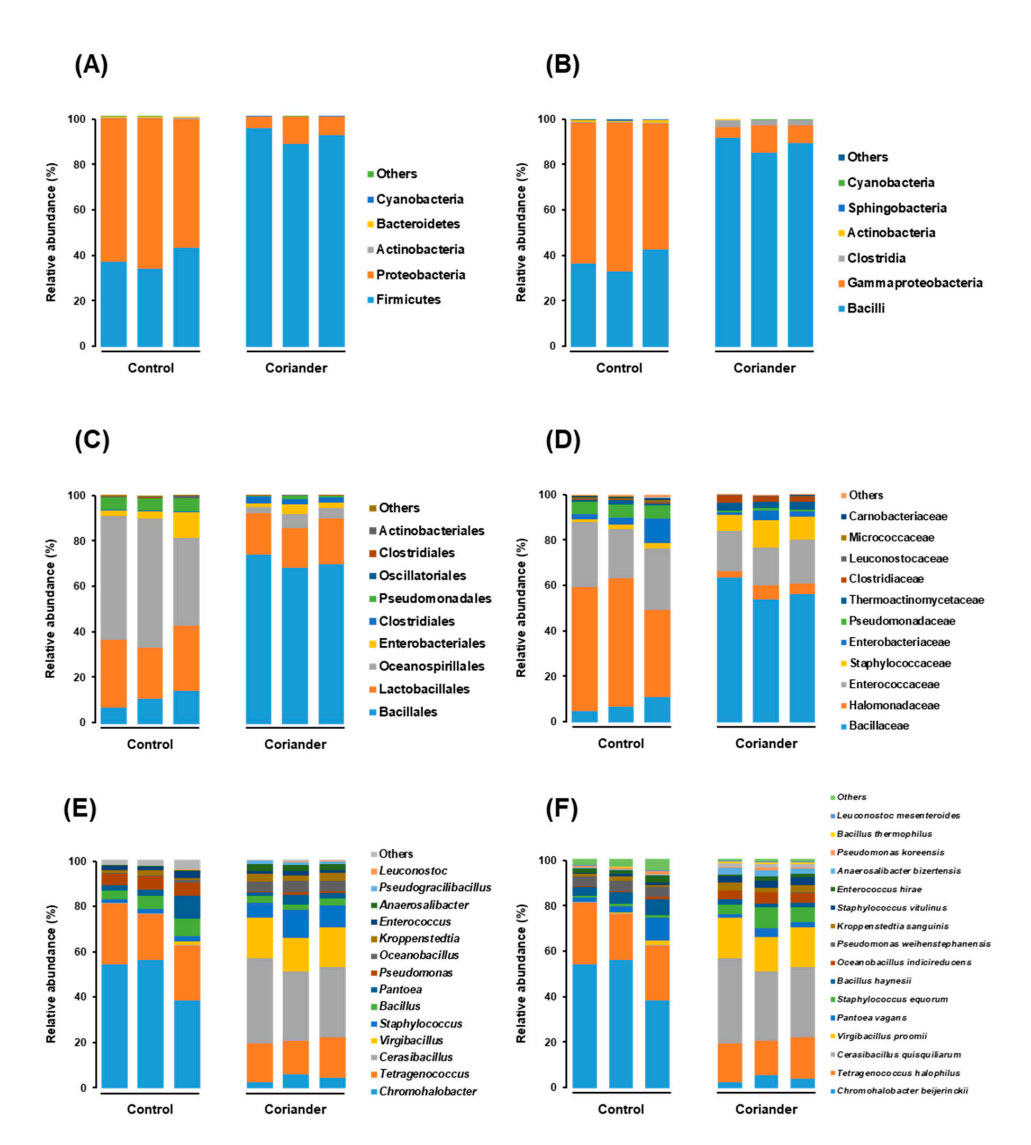
Stacked bar graphs representing the microbiome structure in soy sauce samples prepared by adding coriander, compared to that of control: (**A**) phylum level, (**B**) class level, (**C**) order level, (**D**) family level, (**E**) genus level, and (**F**) species level.

Noticeable differences were observed upon plotting the relative abundance of microbial taxa at different taxonomic levels on stacked bar graphs. At the phylum level, Firmicutes and Proteobacteria were the two major groups in all the samples. Firmicutes was abundant in all the coriander soy sauce samples, with a relative abundance average of 91.78% ± 0.03% compared to 38.03% ± 0.04% in the control group, whereas Proteobacteria was abundant in the control group, with an abundance average of 60.88% ± 0.04% compared to 8.12% ± 0.03% in the coriander group (Figure 3A). The Firmicutes and Proteobacteria in both samples were mainly composed of members of the Bacilli and Gammaproteobacteria classes, respectively (Figure 3B).

At the order level, the main difference was the dominance of the Bacillales and Oceanospiralles in the coriander and control groups, respectively, with corresponding abundance averages of 70.71% ± 0.02% and 49.77% ± 0.08% (Figure 3C). At the family level, the coriander group was dominated by Bacillaceae, Enterococcaceae, Staphylococcaceae, and Halomonadaceae, with average relative abundances of 57.95% ± 0.04%, 17.94% ± 0.01%, 9.55% ± 0.01%, and 4.54% ± 0.02%, respectively. The control group was dominated by Halomonadaceae, Enterococcaceae, Bacillaceae, and Pseudomonadaceae, with average relative abundances of 49.77% ± 0.08%, 25.67% ± 0.03%, 7.63% ± 0.03%, and 5.51% ± 0.00%, respectively (Figure 3D). At the genus level, the coriander group was dominated by *Cerasibacillus, Virgibacillus, Tetragenococcus, Staphylococcus*, and *Chromohalobacter* spp., with average relative abundances of 32.82% ± 0.03%, 16.51% ± 0.01%, 16.48% ± 0.01%, 9.54% ± 0.02%, and 4.44% ± 0.01%, respectively. The control group was dominated by *Chromohalobacter, Tetragenococcus, Bacillus, Pseudomonas*, and *Pantoea* spp., with average relative abundances of 49.54% ± 0.08%, 23.53% ± 0.03%, 5.73% ± 0.01%, 5.51% ± 0.00%, and 5.00% ± 0.04%, respectively (Figure 3E).

**Figure 4:**
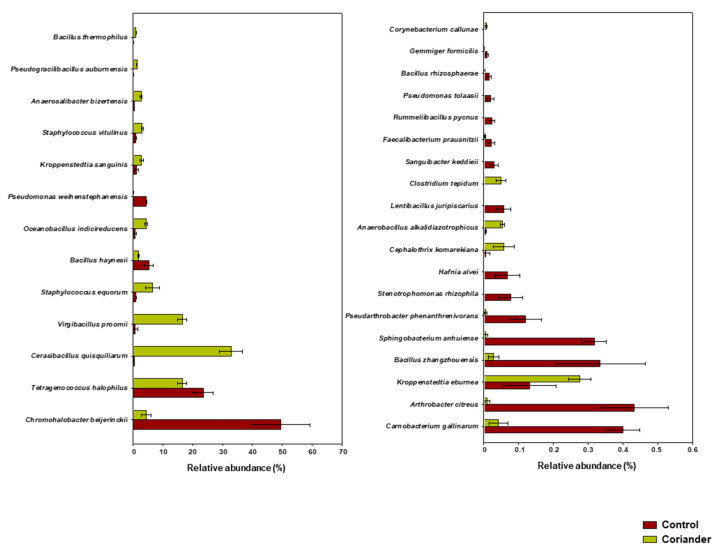
Bacterial species existing with significantly different (*p* < 0.05) relative abundance in soy sauce samples prepared by adding coriander, compared to those in control. Bars represent means and standard deviations.

At the species level, the coriander group was dominated by Cerasibacillus quisquiliarum, Virgibacillus proomii, Tetragenococcus halophilus, Staphylococcus equorum, and Chromohalobacter beijerinckii, with average relative abundances of 32.82% ± 0.03%, 16.50% ± 0.01%, 16.48% ± 0.01%, 6.52% ± 0.02%, and 4.44% ± 0.01%, respectively. The control group was dominated by C. beijerinckii, T. halophilus, Bacillus haynesii, Pantoea vagans, and Pseudomonas weihenstephanensis, with average relative abundances of 49.54% ± 0.08%, 23.53% ± 0.03%, 5.24% ± 0.01%, 5.00% ± 0.04%, and 4.31% ± 0.00%, respectively (Figure 3F). The microbial taxa that existed at significant levels (*p* < 0.05) in the coriander and control soy sauce groups are shown in Figure 4.

**Figure 5:**
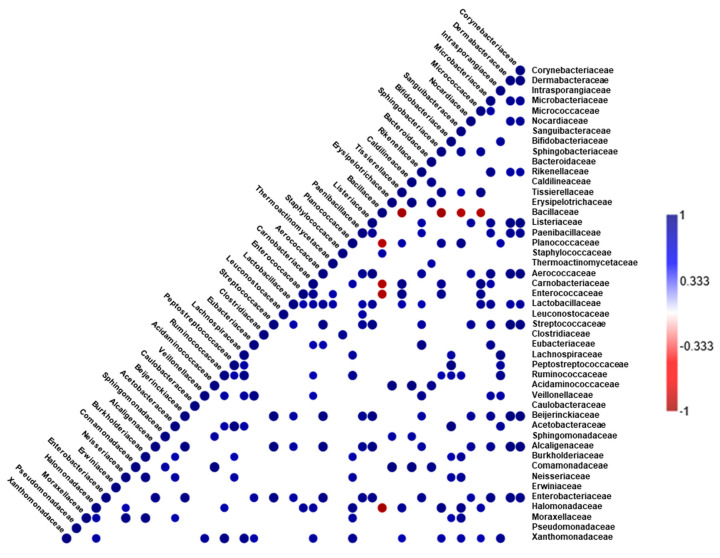
Correlogram representing matrices of Pearson’s correlation coefficient (*r*) between different bacterial taxa in soy sauce samples (*n* = 6), at family level. Only significant (*p* < 0.05) positive (blue) and negative (red) correlations are shown in the graph.

The Pearson’s correlation relationship among the microbial taxa at the family level indicated various positive correlations; only the Bacillaceae family showed significant negative correlation with the Halomonadaceae, Carnobacteriaceae, Enterococcaceae, Planococcaceae, Micrococcaceae, Sanguibacteriaceae, Sphingobacteriaceae, and Tissierellaceae families (Figure 5).

### 3.3. Reduction in the Biogenic Amine Content in Korean Soy Sauce upon Addition of Coriander during Fermentation

**Figure 6:**
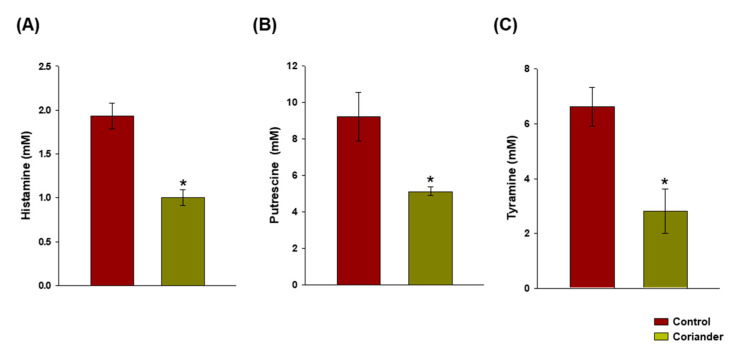
Quantification of biogenic amines, (**A**) histamine, (**B**) putrescine, and (**C**) tyramine, in Korean soy sauce samples prepared by adding coriander during fermentation, compared to those in the control. *: a significant difference at *p* < 0.05.

^1^H NMR quantification of biogenic amines revealed that the contents of histamine, putrescine, and tyramine were significantly (*p* < 0.05) lower in the Korean soy sauce samples prepared by adding coriander during fermentation, compared to those in the control (Figure 6). The percentages of inhibition were 48.03% ± 3.65%, 43.96% ± 3.27%, and 57.74% ± 4.89% for histamine, putrescine, and tyramine, respectively.

## 4. Discussion

Fermented food microbiota are directly associated with the quality and safety aspects of the food, and may directly or indirectly affect human health [29]. Therefore, the ultimate objective for improving food quality is to ensure product safety by determining the microbial composition of fermented foods and analyzing the possibilities for deliberately manipulating the community structure [30]. In this study, the microbiota in Korean soy sauce were investigated, and the effect of coriander on the microbial composition and the content of biogenic amines was studied, with the intention of regulating the fermentation conditions to improve soy sauce quality.

An earlier study using a 16S rRNA PCR-based culture-independent approach indicated that Korean soy sauce microbiota are mainly composed of *Haloanaerobium, Tetragenococcus, Staphylococcus*, and *Bacillus* spp. [9]. A more recent study that utilized a next-generation high-throughput sequencing-based approach—similar to the approach used in the current study—indicated that the halotolerant and halophilic microbes derived from the sea salt added during fermentation—such as *Tetragenococcus, Staphylococcus*, and *Chromohalobacter* spp.—were responsible for Korean soy sauce fermentation [15]. *Tetragenococcus* was found in another study to be related to production of aroma-active and umami taste constituents such as aspartic acid, glutamic acid and alanine, indicating its important role in the fermentation of soy sauce [31]. Consistently, the control soy sauce group in the current study was dominated by halophilic *Chromohalobacter* and *Tetragenococcus* spp., whereas the coriander group was dominated by *Tetragenococcus* spp. and spore-forming Bacillaceae such as *Cerasibacillus* and *Virgibacillus* spp., indicating a clear shift between the soy sauce groups. Spore-forming Bacillaceae can survive extreme environmental conditions—such as high-salt and undernutrition environments—that may kill vegetative bacterial cells [32]. Non-halophilic microbes that survive during the second fermentation step are mainly derived from meju, and may not play a major role in soy sauce fermentation [15].

The significant shift in the microbial community in the coriander soy sauce group can be attributed to the antimicrobial activity of the bioactive compounds present in coriander. Coriander has been shown to contain volatile compounds that may be detrimental to the growth of certain bacterial groups [18]. The essential oils present in coriander have also been shown to exert antimicrobial activity against Gram-negative and Gram-positive foodborne pathogenic bacteria, such as *Escherichia coli, Salmonella typhimurium, Listeria monocytogenes*, and *Staphylococcus aureus* [21]. Moreover, coriander oil shows potent antimicrobial activity against pathogenic *Campylobacter jejuni* [33]. In the current study, there was a clear distinction between the microbial compositions of the soy sauce prepared using coriander and the control, as Firmicutes was dominant in coriander soy sauce, while Proteobacteria was dominant in the control.

The control soy sauce was dominated by *C. beijerinckii* with approximately 50% relative abundance, which dramatically decreased in the coriander soy sauce to less than 5%. *Chromohalobacter beijerinckii* is a psychrophilic, extremely halotolerant Gram-negative bacterium typically found in highly salted environments such as salty beans and herrings [34]. It is known to produce biogenic amines, such as putrescine, histamine, and tyramine, which are low-molecular weight nitrogenous microbial metabolites originating as a result of the decarboxylation of specific amino acids and nitrogen compounds during fermentation [12,35]. The excessive consumption of biogenic amines is associated with adverse toxicological effects [12]. Hence, it is important to monitor the potential microbial producers of biogenic amines in fermented foods and to manage their population.

In a previous study, simultaneous investigation of the metabolites and the bacterial community of Korean soy sauce showed that *Chromohalobacter* was the dominant genus toward the end of the fermentation, and that it closely correlated with the production of biogenic amines, including putrescine [27]. In the current study, the changes in the microbial composition could consistently explain the significant reduction in the levels of biogenic amines (histamine, putrescine, and tyramine) in the coriander soy sauce samples. The presence of histamine, putrescine, and tyramine, as well as other biogenic amines in food, is associated with health risks; owing to their potential toxicity, avoiding their accumulation in foods is advisable [36]. Histamine poisoning is a known risk factor associated with the consumption of histamine-rich foods and could lead to serious allergen-type reactions at high levels [37]. The presence of other types of biogenic amines such as putrescine enhance the toxicity of histamine [38]. In addition, acute and subacute putrescine and tyramine toxicity were confirmed in animal model studies and their consumption in large amounts was linked to dietary-induced migraines and hypertensive crisis [38,39]. Therefore, the significant reduction in *C. beijerinckii* abundance in coriander soy sauce suggests that coriander addition during the fermentation of soy sauce is beneficial as it controls the levels of biogenic amines.

A correlation analysis provided insights into the relationships among the detected microbes, although several other factors—during the fermentation process—should have been considered. The significant negative correlation between Bacillaceae and Halomonadaceae could be attributed to possible antagonistic activity that resulted in a reduction in the relative abundance in the coriander soy sauce group, which showed a higher relative abundance of Bacillaceae, but showed a low abundance of potential biogenic amine producers, i.e., *Chromohalobacter* spp.

The results of this study suggest that adding coriander during the fermentation of Korean soy sauce is beneficial for inhibiting the production of undesirable metabolites. In particular, the levels of the biogenic amines, histamine, putrescine, and tyramine, and the biogenic amine–producing bacterium *C. beijerinckii* were significantly lower in the coriander-supplemented Korean soy sauce compared to those in the control. Further studies that consider other quality and safety aspects related to the addition of coriander during soy sauce fermentation are required.

## Supplementary Materials

The following are available online at https://www.mdpi.com/2304-8158/9/10/1346/s1, Table S1: Results of high-throughput sequencing, representing total bases, read counts, GC%, Q20%, and Q30%.
