# Supplementary material for: Addition of Coriander during Fermentation of Korean Soy Sauce (Gangjang) Causes Significant Shift in Microbial Composition and Reduction in Biogenic Amine Levels
Source: Foods. 2020 Sep 23;9(10):1346. doi: 10.3390/foods9101346 (PMC7598154; doi:10.3390/foods9101346)
Supplement: Supplementary file 1 [file foods-09-01346-s001.pdf]

Table S1. Results of the high throughput sequencing representing the total bases, read count, GC%, Q20% and Q30%

Sample	Total bases	Read count	GC (%)	Q20 (%)	Q30 (%)
SSC1	118032650	254073	54.55	98.76	95.65
SSC2	111544163	240129	54.90	98.75	95.76
SSC3	117945445	253943	54.35	98.86	95.99
SST1	95979498	206738	53.38	98.76	95.54
SST2	114442144	246468	53.35	98.77	95.68
SST3	80305492	172912	53.36	98.74	95.57

SSC, soy sauce control; SST, soy sauce prepared by adding coriander during soy bean fermentation.
